# *Harmonia manillana* (Mulsant), a new addition to Indian Coccinellidae, with changes in synonymy

**DOI:** 10.3897/BDJ.4.e8030

**Published:** 2016-03-25

**Authors:** J. Poorani, Roger G Booth

**Affiliations:** ‡ ICAR-National Research Centre for Banana, Thogamalai Road, Thayanur Post, Trichy, India; § The Natural History Museum, Cromwell Road, SW7 5BD, London, United Kingdom

**Keywords:** *Harmonia manillana*, *Harmonia dunlopi*, new synonymy, Coleoptera, Coccinellidae

## Abstract

**Background:**

*Harmonia dunlopi* (Crotch), a rare lady beetle species, was originally described from 'India' by Crotch (1874). But information on subsequent sightings of this species is absent and it has not been reported by anyone from India and its neighbouring countries ever since its original description. Because of this, Indian records of *H. dunlopi* were suspected to be probably misidentifications of *H. dimidiata* (F.), a species common in northern and northeastern India and also widely distributed in the Oriental region.

**New information:**

A single male specimen of a species collected in recent surveys from Arunachal Pradesh, India, was suspected to be *H. dunlopi*. Comparison of this specimen with the collections at the Natural History Museum, London, confirmed that it belonged to *Harmonia manillana* (Mulsant), hitherto known from Malaysia, Indonesia and the Philippines. *Harmonia manillana* is a highly polymorphic species with many synonyms and based on examination of the type material, the following nomenclatural changes are proposed. *Harmonia dunlopi* was found to be only a color variant of *H. manillana* and hence it is reduced to a junior synonym of *H. manillana* (**syn. nov.**). *Harmonia decussata* (Crotch 1874) is removed from synonymy with *H. manillana* and reinstated as a valid species (**stat. rev.**) and *H. flavomarginata* Bielawski 1968 is a new junior synonym of *H. decussata* (**syn. nov.**). This is the first record of *H. manillana* for India and South Asia. The male genitalia of *H. manillana* are illustrated and compared with those of *H. dimidiata*, the more common Indian species, to facilitate its recognition.

## Introduction

*Harmonia dunlopi* (Crotch 1874) was originally described as *Leis dunlopi* Crotch with "India" as the type locality. It has not been seen in any major Indian collections at the Zoological Survey of India, Kolkata, and the National Pusa Collection at the Indian Agricultural Research Institute, New Delhi. Its name has not been mentioned by any workers from India ever since the original publication. Poorani (2002) included it in her checklist of the Indian fauna of Coccinellidae and indicated that Indian records of *H. dunlopi* could be probably misidentifications of *Harmonia dimidiata* (Fabricius 1781) as suggested by the second author.

During recent surveys in Arunachal Pradesh, northeastern India, a single male specimen of a species thought to be *H. dunlopi* was collected and examined along with several specimens of *H. dimidiata*. This specimen was found to match Malaysian specimens of *H. manillana* (Mulsant) in the collections of the Natural History Museum, London. The male genitalia of the Indian specimen were also found to be identical to those of *H. manillana* illustrated by Bielawski (1962). This is the first record of *H. manillana* for South Asia.

Iablokoff-Khnzorian (1982) listed many synonyms of *H. manillana*, which were also followed by Gordon (1987) and Coutanceau (2008). *Harmonia manillana* is externally quite variable and examination of the type material of these species in the Crotch Collections at Cambridge and the Natural History Museum, London, by RGB indicates there is a need to revisit and change some of these synonymies. The following changes and new synonymies are proposed here: *Harmonia decussata* Crotch (1874) is a valid species and is removed from synonymy with *H. manillana* (**stat. rev.**) and *Harmonia flavomarginata* Bielawski (1968) is a new junior synonym of *H. decussata* (**syn. nov.**). *Harmonia dunlopi* is considered by us as a mere color variant of *H. manillana* and synonymized with *H. manillana* here (**syn. nov.**). A brief diagnostic description is given for *H. manillana* based on the sole Indian specimen examined and the male genitalia of both *H. manillana* and *H. dimidiata* are illustrated to facilitate their identification in the event of any subsequent collection.

## Materials and methods

The specimen examined was part of the collections made in Pasighat and nearby places in the north eastern Indian state of Arunachal Pradesh, in November 2014 and it is deposited in the reference collections of the National Bureau of Agricultural Insect Resources, Bangalore. Photographs of the habitus and the male genitalia were taken using a Leica M205A Stereo microscope. Composite images were generated from image stacks using the software Combine ZP and touched up in Photoshop Elements 11.

The following acronyms are used for the repositories mentioned in this paper:

BMNH - Natural History Museum, London:

MNHUB - Museum für Naturkunde der Humboldt Universität, Berlin

UCCC - University of Cambridge Crotch Collections, Cambridge

## Taxon treatments

### Harmonia manillana

(Mulsant, 1866)

Caria manillana Mulsant 1866: 170 (Type locality: 'Manilla', Philippines; Lectotype, UCCC).-Bielawski 1962: 197.-Gordon 1987: 14 (lectotype designation).
Leis atrocincta Mulsant 1866: 175 (Type locality: 'Manilla', MNHUB).-Crotch 1874: 120 (as var. *atrocincta*).-Coutanceau 2008: 7.
Neda paulinae Mulsant 1866: 203 (Type material: ?MNHUB).-Crotch 1874: 120 (as *Caria paulinae*).-Bielawski 1962: 197.-Iablokoff-Khnzorian 1982: 486.-Gordon 1987: 14.-Coutanceau 2008: 7.
Leis dunlopi Crotch 1874: 121 (Type locality: “India”; Lectotype, UCCC).-Iablokoff-Khnzorian 1982: 486.-Gordon 1987: 14 (lectotype designation).-Coutanceau 2008: 7. **New Synonym.**
Leis cerasicolor Crotch 1874: 121 (Holotype, UCCC).-Iablokoff-Khnzorian 1982: 486.-Gordon 1987: 14.-Coutanceau 2008: 7.
Leis aterrima Crotch 1874: 121 (Holotype, UCCC).-Iablokoff-Khnzorian 1982: 486.-Gordon 1987: 14.-Coutanceau 2008: 7.
Leis papuensis Crotch 1874: 121 (Lectotype, UCCC).-Iablokoff-Khnzorian 1982: 486.-Gordon 1987: 14 (lectotype designation).-Coutanceau 2008: 7.
Leis papuensis var. suffusa Crotch 1874: 121 (Lectotype, UCCC).-Korschefsky 1932: 275.-Gordon 1987: 14 (lectotype designation). **Syn. nov.**

#### Materials

- **Type status:** Other material. **Occurrence:** individualCount: 1; sex: Male; lifeStage: Adult; preparations: Male genitalia; **Taxon:** scientificName: *Harmonia manillana* (Mulsant); kingdom: Animalia; phylum: Arthropoda; class: Insecta; order: Coleoptera; family: Coccinellidae; taxonomicStatus: accepted; **Location:** continent: Asia; country: India; stateProvince: Arunachal Pradesh; municipality: Pasighat; locality: Pasighat College of Horticulture & Forestry; verbatimLocality: College of Horticulture & Forestry; **Identification:** identifiedBy: J Poorani; **Event:** samplingProtocol: Yellow pan trap; eventDate: 2014-11-11/17; year: 2014; month: November; **Record Level:** institutionID: ICAR-NBAIR; institutionCode: NBAIR

#### Description

Length: 6.5 mm. Form (Fig. 1a) hemipherical, strongly convex, dorsum glabrous except head with silvery white hairs around clypeal margin. Dorsal side bright reddish-testaceous, pronotum with a median black macula on posterior margin above scutellum, elytra with 11 black spots, spots on each elytron arranged in a 1-2-2-1/2 pattern, one below anterior margin, two transverse spots arranged just above midline (one lateral and one discal), the second pair positioned around apical third and smaller than the first pair of spots, the last spot sutural and reaching apex; ventral side reddish testaceous except metaventrite medially blackish. Abdominal postcoxal line (Fig. 1b) incomplete with a semi-circular associate line, ventrite 5 apically shallowly emarginate, ventrite 6 slightly more deeply emarginate. Male genitalia (Fig. 1c, d, e, f) with penis guide of tegmen in ventral view (Fig. 1d) basally broadest, progressively narrowed towards a tubularly produced apex, shorter than parameres; parameres with lateral and inner margins covered with dense pubescence. Penis (Fig. 1e, f) with a prominent, stout capsule, penis apex (Fig. 1f) as illustrated. (This description is based on the single specimen examined from India.)

#### Diagnosis

*Harmonia manillana* is externally highly variable and Bielawski (1962) described the nominate form in detail and illustrated the male and female genitalia. In the sole Indian male examined here and the specimens of *H. manillana* from Malaysia at BMNH, on each elytron the spots are characteristically arranged in a 1-2-2-1/2 pattern and the pronotum has a larger median spot. The male genitalia in *H. manillana* are diagnostic. The male genitalia of the Indian specimen fully match the illustrations given by Bielawski (1962) and Iablokoff-Khnzorian (1982).

The nominate form of *Harmonia dunlopi* is very similar to *H. dimidiata* and likely to be confused with it as observed by Bielawski (1962) and Iablokoff-Khnzorian (1982). Crotch (1874) described *H. dunlopi* as having a 1-2-1-1 elytral pattern and observed that an additional sutural spot was present in some specimens. The lone Indian specimen appears to have this extra spot observed by Crotch. The illustration given by Iablokoff-Khnzorian (1982) for *H. dunlopi* also shows a tiny extra spot next to suture in the third row, which corresponds to the Indian specimen. The only specimen of *H. dunlopi* examined by JP in the collections of the Zoological Survey of India, Kolkata, was collected in "Haruhasa Mt. Sambawa" (Indonesia) and identified by A.P. Kapur as *Leis dunlopi* var. nov. (compared with type) (Fig. 2). This specimen was much larger with smaller elytral spots compared to the present example. Further specimens of *H. manillana* from “Haruhasa, Mt. Sambawa” in BMNH were also determined by A.P. Kapur as *dunlopi* var. nov. and are from the same series as the specimen shown in Fig. 2.

*Harmonia dimidiata* (Fig. 3a) is orange-yellow to bright red with a pair of black spots on pronotum, often fused into a single marking with a median emargination and 13 black spots on elytra arranged in a 1-3-2-1/2 pattern. The pronotal spots and apical elytral spot are sometimes absent in some examples (Figs 3a, 4a, d). The elytral color pattern is variable with the spots enlarged (Fig. 4c) or posterior two-thirds of elytra black and anterior portion yellowish, with the humeral black spots present (Fig. 4d) or absent. The abdominal postcoxal lines are incomplete with an associate line as in *H. manillana*. The metaventrite in the Indian specimen of *H. manillana* is almost black, whereas in *H. dimidiata*, the ventral side is reddish testaceous. The male genitalia (Fig. 3b) in *H. dimidiata* are superficially similar to those of *H. manillana*, but the penis guide is distinctly more elongate and narrower than that in *H. manillana* with a rounded apical projection and the penis capsule is elongate with a much longer and narrower outer arm. The parameres in *H. manillana* are stouter and shorter and rather abruptly narrowed in the apical third whereas in *H. dimidiata*, parameres are more or less uniformly wide throughout and apically truncate. The spermatheca and the infundibulum in *H. dimidiata* are illustrated in Fig. 3b. The spermatheca of *H. manillana* illustrated by Bielawski (1962) and Iablokoff-Khnzorian (1982) appears to be different from that of *H. dimidiata* and clearly shows a basal constricted projection of the cornu though the infundibulum appears to be similar in both species. *Harmonia dimidiata* is widely distributed in north, northwestern and northeastern India and several other South and southeast Asian countries in the Oriental region.

#### Distribution

India: Arunachal Pradesh (**new record**); Philippines; Malaysia; Indonesia (Iablokoff-Khnzorian 1982; Coutanceau 2008).

#### Conservation

*Harmonia manillana* appears to be extremely rare in India. Discovery of *H. manillana* from India is important as it could be taken as a confirmation of the type locality of *H. dunlopi* (synonymized here with the former). This is the first record of *H. manillana* from mainland India and its absence in Indian and international collections is an indication that it is probably a very rare species and it probably needs to be listed as such in Indian faunal lists. It is worth noting that this was the only specimen collected along with several specimens of *H. dimidiata* from the same locality.

#### Taxon discussion

Two species, *Harmonia decussata* (Crotch 1874: 161) and *H. flavomarginata* Bielawski (1968), are wrongly synonymized with *H. manillana* by Iablokoff-Khnzorian (1982), and are valid names. Coutanceau (2008) appears to have followed Iablokoff-Khnzorian in his checklist of world species of *Harmonia*, where both are listed as synonyms of *H. manillana*. Crotch (1874) described *Callineda decussata* from various localities and the syntype series in UCCC and BMNH is very mixed, but none is *H. manillana*. The lectotype of *C. decussata*, designated by Gordon (1987), is here confirmed as a valid species, *Harmonia decussata* (Crotch, 1874) (**stat. rev.**). This lectotype was examined by RGB in 1989 and found to represent the same species that Bielawski (1968) described as *Harmonia flavomarginata* (**syn. nov.**). This also appears to be the same species that Chazeau (1989) described as *Harmonia incognita*, but a formal synonymy of the latter must await further study.

Crotch (1874) listed *Leis suffusa* as a variety of *L. papuensis* (now a synonym of *H. manillana*) and Korschefsky (1932) listed it as an aberration of *papuensis* in his catalogue. Gordon (1987) designated a lectotype for *L. suffusa* and mentioned that it appeared to be a synonym of *H. manillana* though it was not formally designated so by Iablokoff-Khnzorian (1982). We formally synonymize *L. suffusa* with *H. manillana* here (**syn. nov.**).

Iablokoff-Khnzorian (1982) described both *H. manillana* and *H. dunlopi* with rather inadequate / poor illustrations. He illustrated the adult and the female genitalia of *H. dunlopi* and indicated that it could be probably synonymous with *H. dimidiata*. He also mentioned that the specimen he examined from the Crotch Collection at the University of Cambridge (UCCC) was a holotype. However, Crotch (1874) clearly mentioned about additional specimens. Gordon (1987) also noted this when he designated a lectotype from Crotch's material at UCCC.

Crotch’s original description of *Leis dunlopi* listed material from India (Dublin, B.M.). Gordon (1987) noted the locality datum on the Lectotype as India. However, the specimen in BMNH, a paralectotype, bearing Crotch’s name label in his own handwriting “Dunlopi ns” is from the Philippines and not India, and this specimen has the color pattern as shown in Fig. 1a. Crotch’s Lectotype was examined by RGB in 1988/89 and the paralectotype was examined again during the preparation of this paper, enabling the synonymy of *H. dunlopi* with *H. manillana* to be confirmed.

## Supplementary Material

XML Treatment for Harmonia manillana

## Figures and Tables

**Figure 1a.**
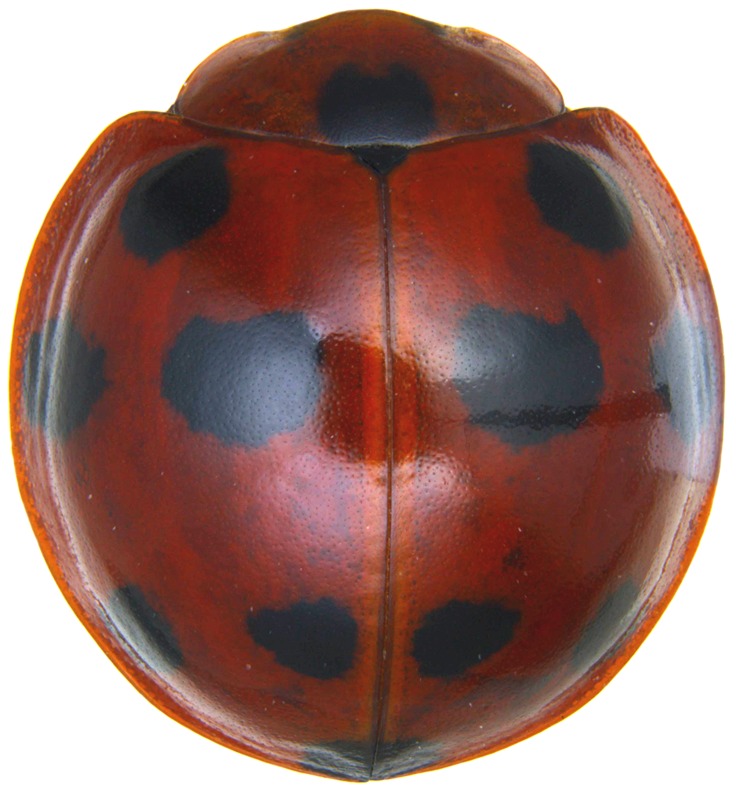
Adult, dorsal habitus

**Figure 1b.**
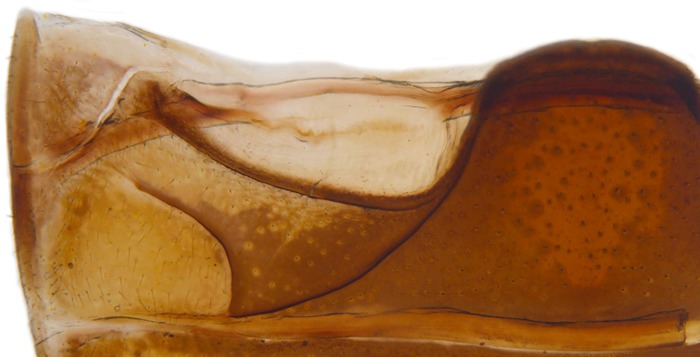
Abdominal postcoxal line

**Figure 1c.**
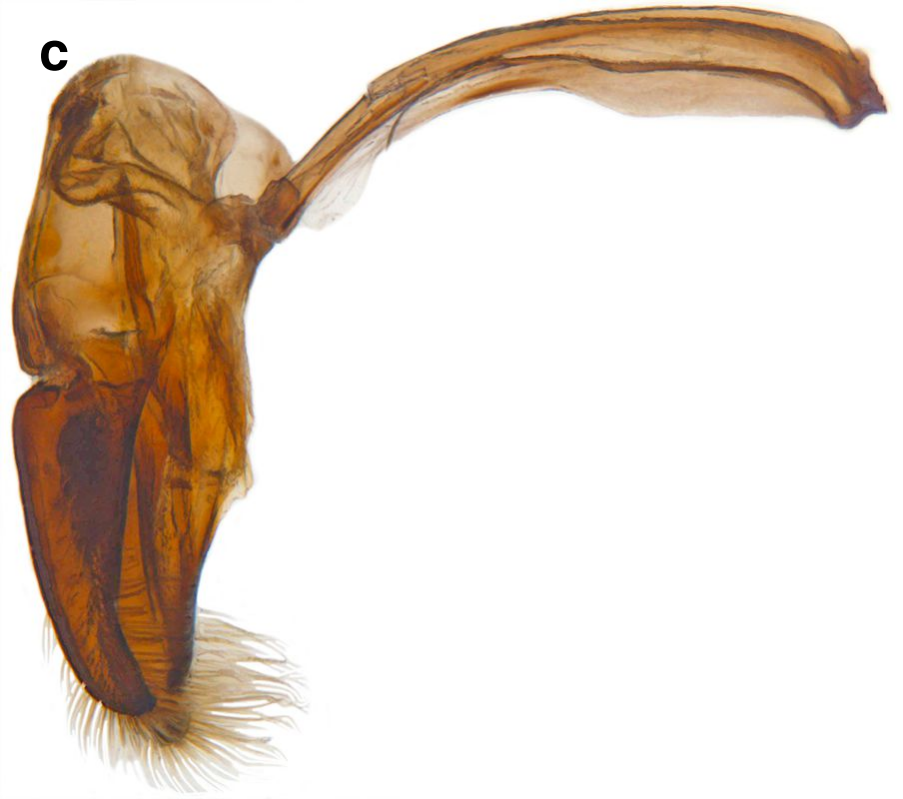
Male genitalia: Tegmen, lateral view

**Figure 1d.**
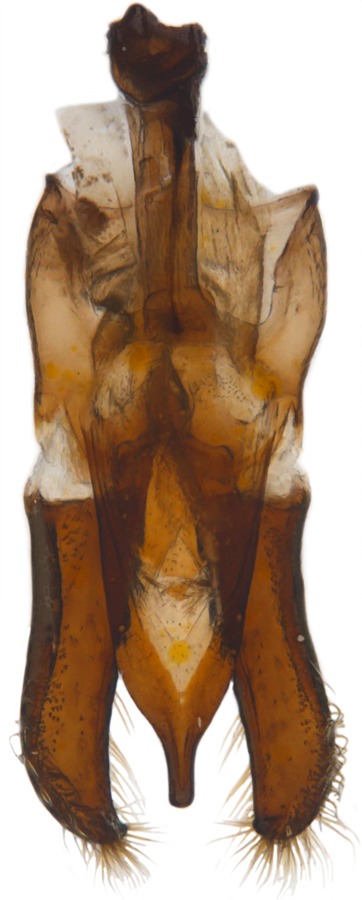
Male genitalia: Tegmen, ventral view

**Figure 1e.**
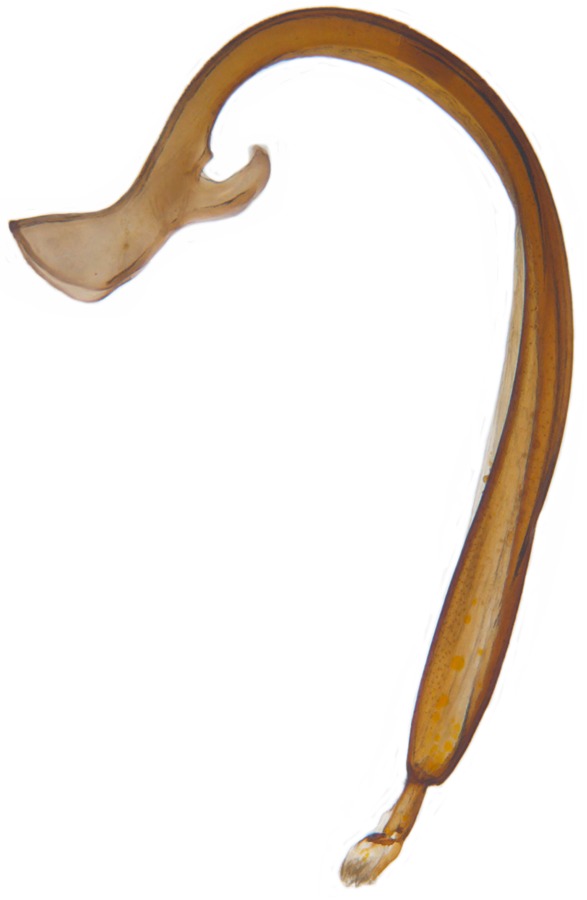
Male genitalia: Penis

**Figure 1f.**
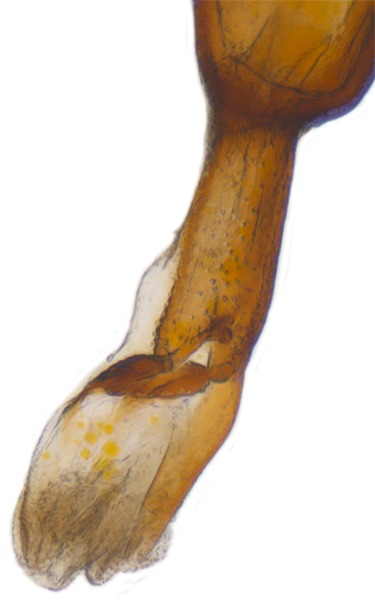
Male genitalia: Penis apex

**Figure 2a.**
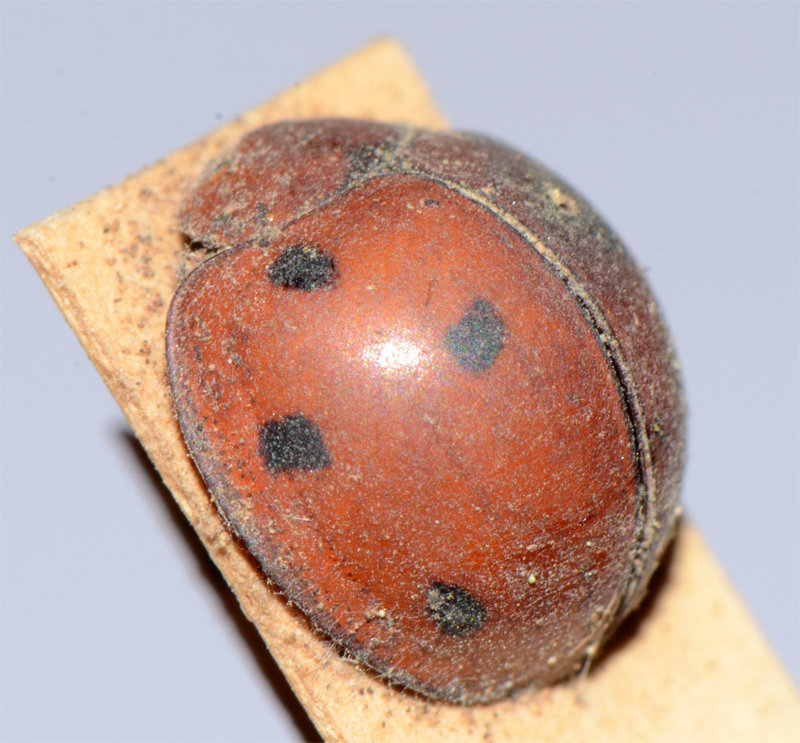
Adult, lateral view

**Figure 2b.**
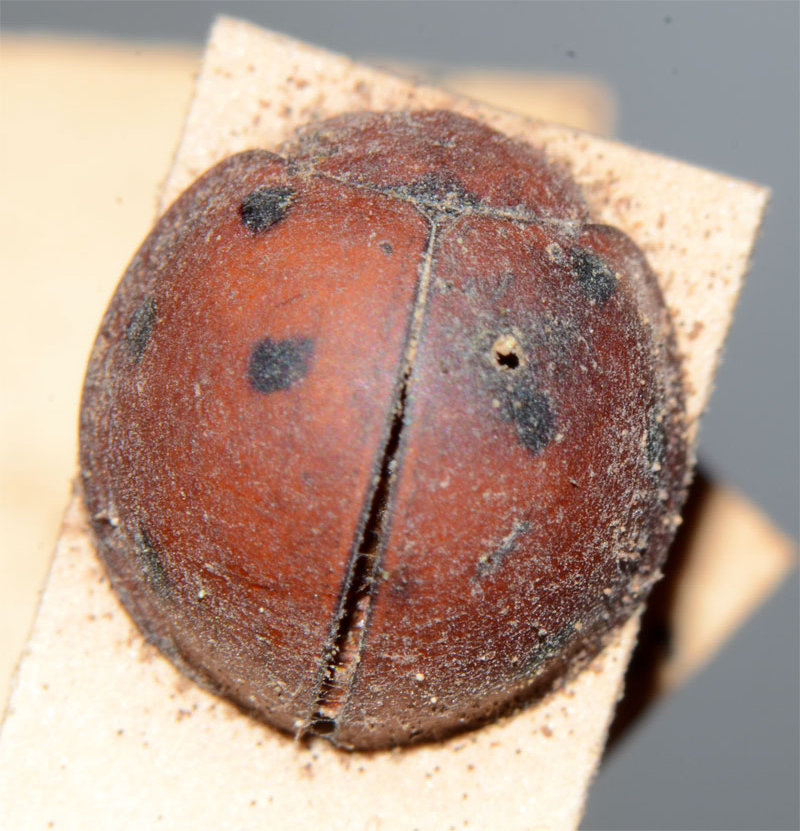
Adult, dorsal view

**Figure 3a.**
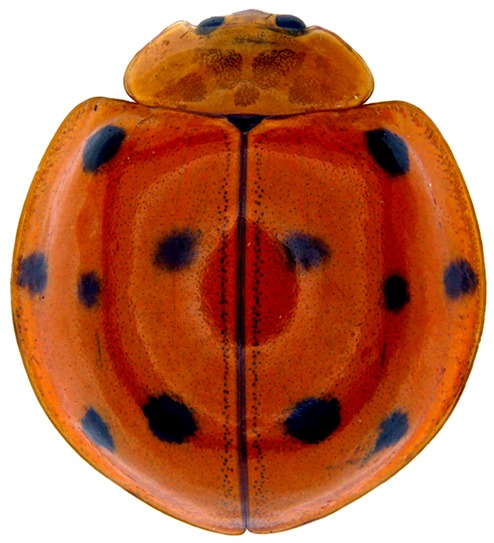
Adult, dorsal view

**Figure 3b.**
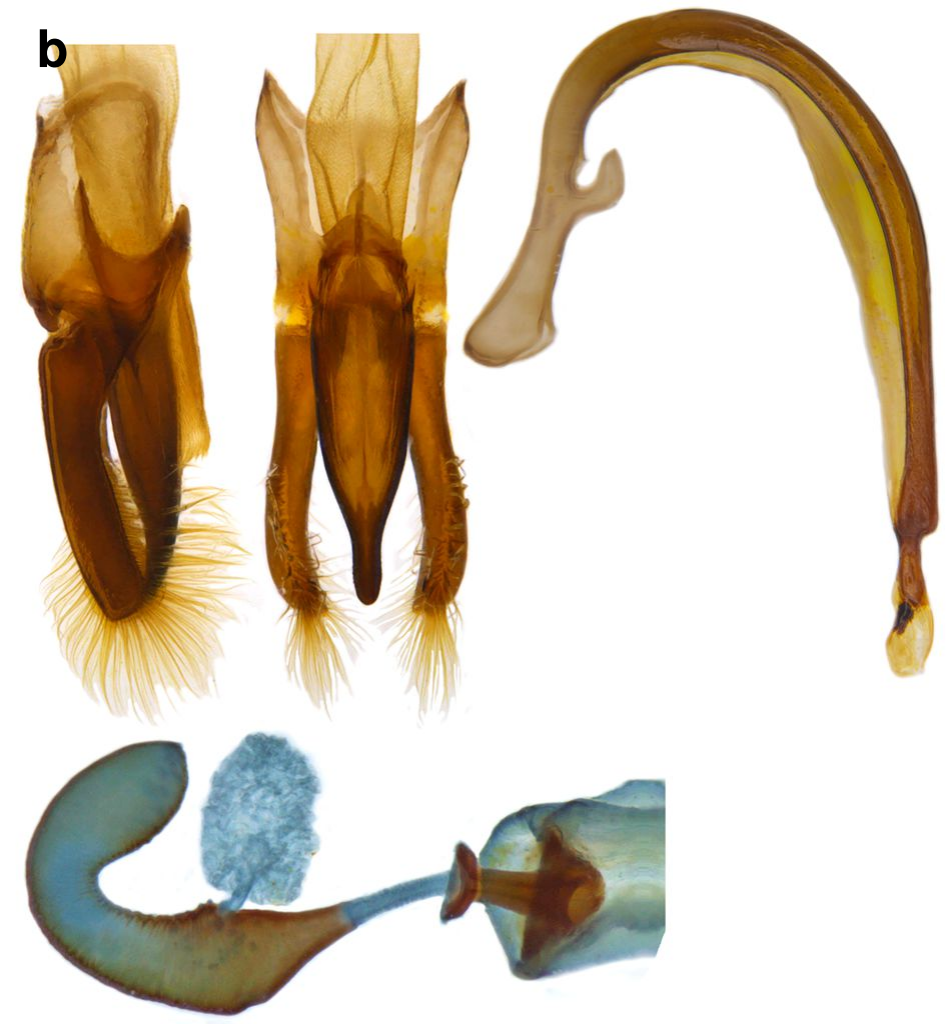
Genitalia

**Figure 4a.**
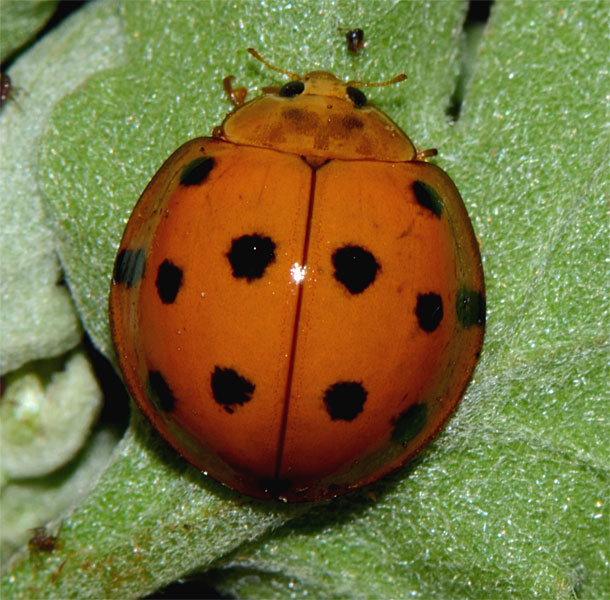
Without pronotal spots

**Figure 4b.**
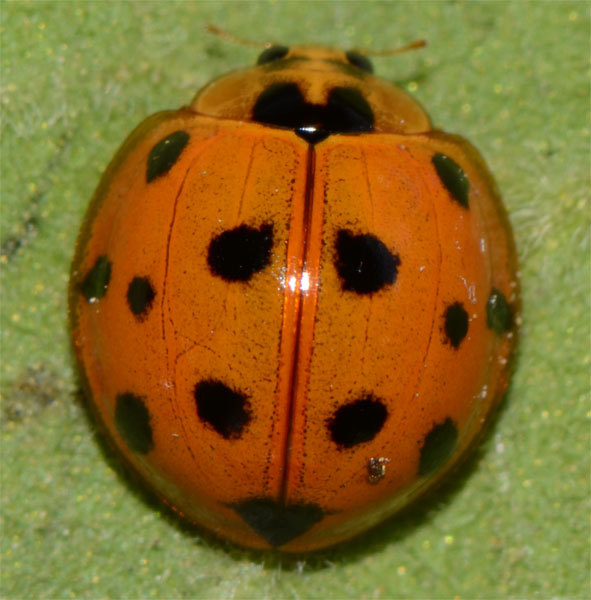
With macula on pronotum

**Figure 4c.**
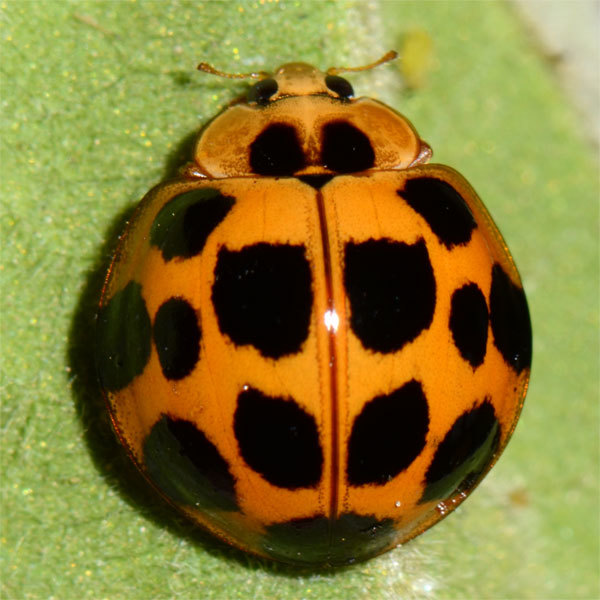
With enlarged spots

**Figure 4d.**
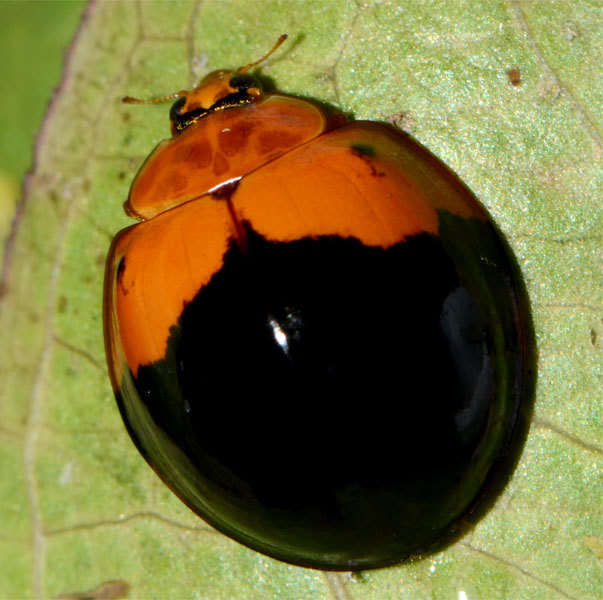
Without pronotal and elytral spots
